# A novel RING finger in the C-terminal domain of the coatomer protein α-COP

**DOI:** 10.1186/s13062-015-0099-9

**Published:** 2015-12-14

**Authors:** Gurmeet Kaur, Srikrishna Subramanian

**Affiliations:** CSIR-Institute of Microbial Technology (IMTECH), Sector 39-A, Chandigarh, 160036 India

**Keywords:** Cellular transport, Coat protein, Coated vesicles, Tethering complex, Zinc finger

## Abstract

The C-terminal domain of α-COP, an essential subunit of the COPI coatomer complex, is composed of an all α-helical region and a small β-sheet domain. We show that this β-sheet domain is a Really Interesting New Gene (RING)-like treble clef zinc finger. The zinc-binding residues are substituted by other aminoacids in many homologs including the structurally-characterized proteins from *Saccharomyces cerevisiae* and *Bos taurus*. This RING-like domain is possibly related to those of other vesicle membrane-associated complexes, such as CORVET, HOPS and SEA, and likely mediates interactions with Dsl1p and assist in coat oligomerization.

**Electronic supplementary material:**

The online version of this article (doi:10.1186/s13062-015-0099-9) contains supplementary material, which is available to authorized users.

## Finding

**Fig. 1:**
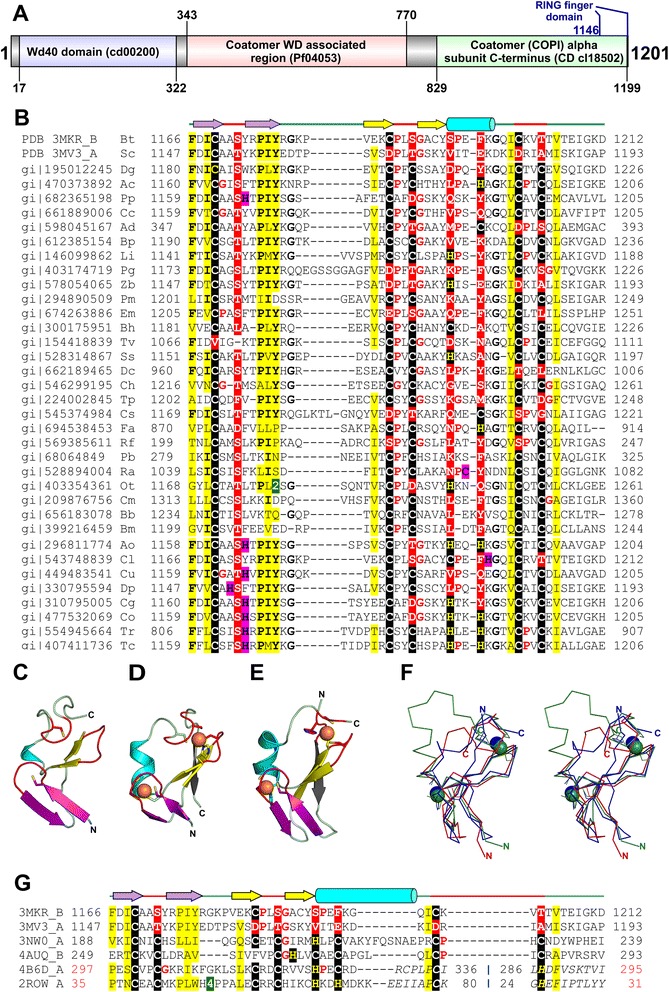
Structure and sequence comparison of α-COP and other RING fingers. **a** Domain diagram of *S. cerevisiae* α-COP. Domain boundaries were obtained by referring to CDD (Additional file 1). Position of the manually delineated RING finger domain is indicated in blue. **b** Structure-based multiple sequence alignment of RING domain from α-COP homologs. **c** RING domain of α-COP (PDBid 3MKR_B) (**d**) A classical RING finger domain from Baculoviral IAP repeat-containing protein 7 (PDBid 4AUQ_B) (**e**) A circularly permuted RING finger from Rac GTPase-activating protein 1 (PDBid 4B6D_A). The RING fingers are colored alike, zinc-binding knuckles: red, primary β-hairpin: yellow, α-helix: cyan, zinc knuckle containing β-hairpin: violet, the additional β-strand: gray, zinc ion: orange sphere. The N- and C-termini are labeled ‘N’ and ‘C’, respectively. Side chains of metal-chelating residues are shown in stick representation. **f** Stereo diagram of the superimposition of the α-COP RING finger (PDBid 3MV3_A; colored red), RING finger domain of Non-SMC element 1 homolog (PDBid 3NW0_B; colored green) and C1 domain of Rac GTPase-activating protein 1 (PDBid 4B6D_A; colored blue). The C_α_ backbone trace is shown for each structure, and bound zinc ions are shown as spheres in the respective colors. **g** Structure-based sequence alignment of RING domain of α-COP (PDBid 3MKR_B, 3MV3_A), RING finger domains (PDBid 3NW0_A, 4AUQ_B) and circularly permuted RING fingers (PDBid 4B6D_A, 2ROW_A). For panels (**b**, **g**) PDBid/Gene identification (gi) number, the first and the last residue numbers of the sequences depicted in the alignment are indicated for each sequence. The secondary structure elements of the RING finger domain are indicated above the alignment. The potential metal-binding ligands are boxed in black, non-metal-binding residues at the same position are boxed in red and adjacent residues that could potentially chelate metal ion are boxed in magenta. Conserved residues are in bold. Small aminoacids (Gly, Pro) in the vicinity of the zinc-chelating ligands are colored red. Uncharged residues (all aminoacids except Asp, Glu, Lys and Arg) in mostly hydrophobic sites are highlighted yellow. Some insertions are not shown and the number of omitted residues is specified by numbers boxed in green. The sequence numbers in the region of permutation in panel (**g**) are shown in red and regions of circular permutation are separated by a ‘**|**’ symbol. In panel (**b**) the PDBid/gi number is followed by organism name abbreviation as follows: Bt- *Bos taurus*, Sc- *Saccharomyces cerevisiae*, Dg- *Drosophila grimshawi*, Ac- *Acanthamoeba castellanii* str. *Neff*, Pp- *Pseudogymnoascus pannorum* VKM F-4516 (FW-969), Cc- *Coffea canephora*, Ad- *Auricularia delicata* TFB-10046 SS5, Bp- *Bathycoccus prasinos*, Li- *Leishmania infantum* JPCM5, Pg- *Puccinia graminis* f. sp. *tritici* CRL 75-36-700-3, Zb- *Zygosaccharomyces bailii* ISA1307, Pm- *Perkinsus marinus* ATCC 50983, Em- *Echinococcus multilocularis*, Bh- *Blastocystis hominis*, Tv- *Trichomonas vaginalis* G3, Ss- *Schizosaccharomyces cryophilus* OY26, Dc- *Diaphorina citri*, Ch- *Chondrus crispus*, Tp- *Thalassiosira pseudonana* CCMP1335, Cs- *Coccomyxa subellipsoidea* C-169, Fa- *Fonticula alba*, Rf- *Reticulomyxa filose*, Pb- *Plasmodium berghei* ANKA, Ra- *Rozella allomycis* CSF55, Ot- *Oxytricha trifallax*, Cm- *Cryptosporidium muris* RN66, Bb- *Babesia bigemina*, Bm- *Babesia microti* strain RI, Ao- *Arthroderma otae* CBS 113480, Cl- *Columba livia*, Cu- *Cucumis sativus*, Dp- *Dictyostelium purpureum*, Cg- *Colletotrichum graminicola* M1.001, Co- *Colletotrichum orbiculare* MAFF 240422, Tr- *Trypanosoma rangeli* SC58, Tc- *Trypanosoma cruzi marinkellei*

The cytoplasmic coat protein I (COPI) complex, which coats the vesicles involved in retrograde transport of proteins from the Golgi apparatus to the endoplasmic reticulum (ER), and in intra-Golgi protein trafficking, comprises of a coatomer and ADP-ribosylation factor 1 (ARF) GTPase [1–3]. The coatomer is a heptameric protein complex with two distinct subcomplexes: an inner cargo-binding heterotetrameric F-subcomplex consisting of β-, γ-, δ- and ζ-COP, and an outer cage-forming heterotrimeric B-subcomplex consisting of α-, β’- and ε-COP. α-COP subunit is a ~1200-residue protein with a N-terminal β-propeller domain (~600-residues; PDBid 4J8G_A), an α-helical region (~200-residues; partial structure in PDBid 3MKQ_B), an unstructured region (~100-residues) and a C-terminal domain (CTD; ~300-residues; PDBid 3MKR_B from *Bos taurus*; PDBid 3MV3_A from *Saccharomyces cerevisiae*) (Fig. 1a) [4–7]. The CTD of α-COP adopts a ‘U’-shaped structure, where an initial stack of 11 α-helices is followed by a β-sheet domain that constitute the descending and the ascending arm of the ‘U’, respectively [6, 7].

The C-terminal β-sheet domain of α-COP is necessary for its interaction with- and incorporation of- ε-COP into the coatomer, for maintaining normal levels of ε-COP, and has also been shown to interact with the β’-subunit [8]. The yeast *ret1-3* allele of α-COP (S1188F mutation in the β-sheet domain) causes instability of α-COP and the coatomer, defects in cellular transport and temperature sensitivity in the organism [8–10]. The CTD has also been suggested to play a role in mediating interactions with Dsl1p subunit of the Dsl1 tethering complex on the ER membranes and assist in the fusion of COPI-coated vesicular membrane with the target membrane [7, 10].

Currently, Pfam [11] and Conserved Domain Database (CDD) [12], co-classify the complete stretch of α-COP CTD (Pfam: PF06957; CDD: cl18502). We show that the β-sheet region of α-COP CTD (PDBid 3MKR_B, residues 1164–1212; PDBid 3MV3_A, residues 1145–1193) is a Really Interesting New Gene (RING)-like binuclear treble clef zinc finger domain. However, the β-sheet domain of α-COP CTD has undergone structural changes and lost its ability to chelate zinc in many organisms.

A multiple sequence alignment of the β-sheet domain of representative α-COP sequences, obtained after PSI-BLAST [13] and JackHMMER [14] sequence-similarity searches (Additional file 1), reveals putative zinc-chelating residues in homologous sequences at expected structural positions of a RING-like domain (Fig. 1b). We observe strong conservation of aminoacids coordinating the second metal ion. Residues coordinating the first metal ion are substituted by other aminoacids in many organisms, except for the first cysteine that is highly conserved (Additional file 2). Of the sequences retrieved, α-COP β-sheet domain would likely bind two metal ions in *Trypanosoma cruzi* (gi|407411736) and *Colletotrichum orbiculare* (gi|477532069) (Fig. 1b). Other conserved hydrophobic and aromatic residues are present in the β-strands and the extended loop region of the domain.

Consistent with the observed pattern of conservation for the zinc-coordinating residues in the α-COP β-sheet domain, which is particularly high for the second metal-coordinating site, sequence-profile-similarity-based searches using HHpred [15] and FFAS [16] find fortuitous matches to many zinc ribbon domains (for example, HHpred, finds TFIIEα zinc ribbon, PDBid 1VD4_A, E-value = 0.014). These zinc ribbons are not homologous to the RING domain of α-COP and lack sequence similarity outside of the metal-coordinating sites. These searches also find matches to RING domains, although with lower statistical significance. HHpred finds matches to Pfam Prokaryotic RING finger family 1 (PF14446, E-value = 0.046), and FFAS finds Non-structural maintenance of chromosomes (SMC) element 1 homolog (PDBid 2CT0_A, Score = −5.28), putative zinc-finger of transcription factor IIIC complex (PF12660, Score = −5.82) and Rho-associated protein kinase 2 (PDBid 2ROW_A, Score = −7.64).

Structurally, the β-sheet domain of α-COP consists of two β-hairpins, a small α-helical turn, and a loop with a tight turn (Fig. 1c). Albeit minor structural variations, this topological arrangement resembles a RING-like fold (Fig. 1d) [17–20]. Classical (mononuclear) treble clefs comprise of a zinc knuckle (a non-canonical turn with the sequence CPXCG), a primary β-hairpin and an α-helix, with the zinc-binding site formed by the zinc knuckle and N-terminal of the α-helix [17]. In the RING-like cross-braced binuclear treble clefs, a second zinc ion is coordinated by ligands contributed by the turn of the primary β-hairpin and an extended knuckle-like region (also referred to as a ‘squiggle’) after the α-helix (Fig. 1d, e) [18, 19]. Most RING fingers have an additional C-terminal β-strand that forms a three-stranded β-sheet with the primary β-hairpin (Fig. 1d). The second metal-binding site is structurally similar to rubredoxin-like zinc ribbons [17, 21].

In the β-sheet domain from structurally-characterized α-COP, the canonical α-helix of the treble clef is reduced to a single turn, and the additional β-strand seen in RING fingers is absent. None-the-less, automated structure-similarity searches with the β-sheet domain of α-COP using Dali [22] retrieved matches to other treble clefs, with top-scoring matches to RING fingers. The retrieved matches included RING domain of E3 ubiquitin-protein ligase EL5 (PDBid 1IYM_A, Z-score = 3.7, RMSD = 2.4 Å, nali = 42), U-box domain of pre-mRNA splicing factor PRP19 (PDBid 2BAY_D, Z-score = 3.4, RMSD = 2.0 Å, nali = 40) and C1 domain of Rac GTPase-Activating Protein 1 (PDBid 4B6D_A, Z-score = 3.8, RMSD = 2.4 Å, nali = 40) (Fig. 1e). We could manually superimpose the β-sheet domain of α-COP (PDBid 3MV3_A) and the RING finger of Non-SMC element 1 homolog (PDBid 3NW0_B) with an RMSD of 1.3 Å over 35 pairs of backbone C_α_ atoms (Fig. 1f, g). Automated pairwise protein structure superimposition using TM-align [23] and Fr-TM-align [24] produced similar results with a TM-score >0.5, indicating a fold level similarity of α-COP β-sheet domain with other RING domains.

Zinc finger domains occur in several proteins of cellular transport pathways in a variety of functional contexts (Additional file 2). Interestingly, we find that subunit proteins of few other vesicle membrane-associated complexes such as Vps8, Vps11, Vps18 and Vps39 of the CORVET (class C core vacuole/endosome tethering) and the late endosomal/lysosomal HOPS (homotypic fusion and protein sorting) complexes [25], and Sea4 of the SEA (Seh1-associated) complex [26] share similar domain architectures, including the β-propeller, the α-solenoid and the RING finger, with the α-COP. Additionally, Sea2 and Sea3 of the SEA complex also have a RING finger and a β-propeller but lack the α-solenoid region [26]. All the membrane-associated coating- and trafficking-related and nuclear pore complexes are suggested to have diverged from a progenitor ‘protocotomer’ which was present in the first common eukaryotic ancestor (FECA) [27, 28]. Although our sequence-similarity searches with the RING domain of α-COP were not able to retrieve the RING domains of these proteins, based on common domain architectures and similar biological pathways where they function, the RING domains in all these proteins plausibly share a distant evolutionary relationship. The RING domain in the proteins of CORVET and HOPS is important for endolysosomal transport by mediating fusion and docking of vesicles at vacuoles, mediating interaction with other proteins and heterodimerization [29–31]. The RING domain of Vps18 also mediates ubiquitination *in vitro* [32, 33].

Currently, no specific function is attributed to the RING finger of α-COP. It is plausible that like bonafide RING domains, the RING finger of α-COP may also possess E3-ubiquitin ligase activity that might affect the assembly and function of the coatomer as proposed for SEA-complex proteins [26]. The C-terminal 19 residues of α-COP, covering a part of the RING domain, are known to enhance its interaction with Dsl1p [10]. Mediating protein-protein interactions being one of the primary functional attributes of RING fingers [19, 34], this domain in α-COP could likely play a role in interacting with Dsl1p to effectuate the fusion of the COPI-coated vesicles with the ER membrane.

The C-terminal 19 residues of α-COP have also been suggested to be involved in dimerization and oligomerization when not interacting with Dsl1p [10]. Moreover, the specific regions of the various coatomer subunits that mediate oligomerization during COPI cage formation are not precisely known [6, 35, 36]. Given the known role of RING domains in homodimerization and macromolecular assemblages in various contexts [20], we speculate that the RING finger of α-COP could assist in coat oligomerization. Indeed, a recent study reporting the cryo-electron microscope structure of the COPI coat (PDBid 5A1V) also suggests that the CTD of α-COP is one of the flexible connecting domains involved in oligomerization of coat units (triads) [37]. In conclusion, the detection of a novel RING finger in the COPI complex may aid in gaining additional insights into the function and mechanism of the COPI-mediated vesicle transport system.

## Response to reviewer and editor-in-chief’s comments on the previous version of the manuscript

### Reviewer 1

Dr. Ramanathan Sowdhamini

Report form: Kaur and Subramanian report the presence of a RING finger domain in the C-terminal domain of coatomer protein, α-COP, after a detailed sequence and structural analyses. α-COP forms one of the subunits in the heptameric assembly of cytoplasmic coat protein I. This assembly is present on Golgi apparatus and is responsible for Golgi-to-endoplasmic-reticulum trafficking. Previous reports on structure determination by other groups have mainly concentrated on the quaternary arrangements, interprotomer interactions and overall domain architectures of individual subunits. The putative connections of the C-terminal region of α-COP, discussed through sequence and structural investigations, are compelling. Indeed, such RING fingers have already been reported in analogous biological assemblies like vacuole/endosome tethering and homotypic fusion-protein sorting complexes, thereby providing a strong evolutionary backup for this argument. For all these reasons, I would recommend acceptance of the paper. However, I would like to suggest few changes in the presentation of the work and results before it is ready for publication. These six points are covered below.

1. line 53: I think the authors mean 3MKR, not 3MRK.

Response: *The PDBid has been corrected.*

2. line 89: The premise in which the connections were realized needs to argued out well and subsequently it needs to be referred as ‘RING domain in the C-terminal region of α-COP’. Currently, already there is a mention that the RING finger domain of α-COP is the query. Rather, a better argument is to say that when the C-terminal domain of α-COP was used as a query and aligned with homologues, quite unexpectedly, several homologues were observed to contain a zinc-finger domain. In particular, you may also point out homologues which are annotated to contain a RING-finger domain.

Response: *The suggested change has been included in the present version of the manuscript. None of the α-COP homologs retrieved during our analysis were previously annotated to contain a RING finger domain.*

3. line 135: Do these searches first pick up zinc ribbons and then ring fingers? If so, it will be nice to mention score for zinc ribbons as well.

Response: *We have included the scores for one of the zinc ribbon domain retrieved in the HHpred search.*

4. line 170: The rigid-body superposition needs to be discussed before the visual and more subjective comparisons.

Response: *We have discussed the Dali results before the manual structure analysis.*

5. line 247: “This RING finger has lost its ability to bind to zinc ions …” This phrase appears too speculative since there are atypical zinc-chelating residues (like 3His-Asp as in adiponectin receptors (http://www.nature.com/nature/journal/v520/n7547/fig_tab/nature14301_SF4.html), where a water molecule participates to stabilize zinc binding). There could also be compensatory chelating sites owing to oligomerization. Hence, it may be better to state 'This RING finger has three of the four classical Zn-binding residues mutated' or ‘Sequence analysis suggests that this RING finger may not have zinc-binding function …’

Response: *These lines have been edited in the revised manuscript.*

6. line 276: Specific reference to PYMOL visualization maybe avoided.

Response: *We have retained the reference to PyMOL in the methods section in the Additional file 1 as we used it extensively for doing manual structure analysis.*

Dr. Eugene Koonin

The finding that you report clearly is valid and of certain importance. At the same time, it is highly specific, so I think you made the right choice of describing your finding in a Discovery Note. However, the present manuscript is far too long and detailed for this format, and this could, I believe, discourage Editorial Board members from handling the submission. My suggestion is that, if you remain interested in Biology Direct as the publication venue, you substantially shorten the manuscript by dropping non-essential details and references.

Response: *We have edited the manuscript to suit the submission criteria for a Discovery Note.*

## Additional files

Additional file 1:
**Methods.** Methods employed in this study (DOCX 32 kb) Additional file 2:
**Supplementary results.** Additional supportive evidence in favor of the results presented (DOCX 31 kb)

## Abbreviations

COP: Cytoplasmic coat protein
RING: Really Interesting New Gene
CORVET: Class C core vacuole/endosome tethering
HOPS: Homotypic fusion and protein sorting
SEA: Seh1-associated
ER: Endoplasmic reticulum
ARF: ADP-ribosylation factor
CTD: C-terminal domain
CDD: Conserved domain database
SMC: Non-structural maintenance of chromosomes
FECA: first common eukaryotic ancestor
PDB: Protein Data Bank
