# Supplementary material for: A novel RING finger in the C-terminal domain of the coatomer protein α-COP
Source: Biol Direct. 2015 Dec 14;10:70. doi: 10.1186/s13062-015-0099-9 (PMC4678705; doi:10.1186/s13062-015-0099-9)
Supplement: Additional file 1: — Methods. Methods employed in this study (DOCX 32 kb) [file 13062_2015_99_MOESM1_ESM.docx]

**A novel RING finger in the C-terminal domain of the coatomer protein α-COP**

Gurmeet Kaur, Srikrishna Subramanian

**Methods**

Sequences similar to the β-sheet domain of α-COP (PDBid 3MKR_B; residues 1166-1212) were retrieved using PSI-BLAST [[1](#_ENREF_1)] run against the NCBI non-redundant protein sequence database (NR; Dec 25, 2014; 54,183,042 sequences; 19,531,459,180 total letters). A cut-off threshold E-value=0.001 was used for iterative PSI-BLAST searches run until convergence.

Additional sequence similarity searches initiated with the β-sheet domain of α-COP (PDBid 3MKR_B; residues 1166-1212) were performed using JackHMMER program from the HMMER3 package (against: NR version 2014-06-17; E-value threshold of 0.001) [[2](#_ENREF_2)], FFAS server [[3](#_ENREF_3)] (against PDB, Pfam and SCOP databases) and the HHpred server [[4](#_ENREF_4)] (against: PDB70_27Dec14 and PfamA_27.0, using MSA generation method HHblits run for 5 iterations, E-value threshold of 0.001).

Sequences similar to the β-sheet domain of α-COP retrieved in the PSI-BLAST and JackHMMER searches were aligned using the ClustalW multiple alignment tool [[5](#_ENREF_5)] within the BioEdit software package (version 7.2.2) [[6](#_ENREF_6)] with default parameters. The multiple sequence alignment (MSA) was further manually corrected using structure-alignment as a guide. Cd-hit tool [[7](#_ENREF_7)] was used to cluster the extracted sequences at 40% sequence identity cut-off. The MSA (**Figure 1B**) contains the sequences of structurally-characterized α-COP β-sheet domain (from *B. taurus* and *S. cerevisiae*), representative sequences from the PSI-BLAST search (with less than 40% sequence identity to each other) and few manually selected remote homologs obtained in JackHMMER search.

TM-align, Fr-TM-align, and Dali were used to evaluate the structural similarity of the β-sheet domain of α-COP with other proteins [[8-10](#_ENREF_8)]. The structures of the β-sheet domain of α-COP (PDBid 3MKR_B, 3MV3_A) were used as queries for automated structure similarity searches. Visualization and comparison of structures were done in the molecular visualization program PyMOL. The pair fitting command of PyMOL was used for the manual superimposition of the structures.

Domain Graph (DOG) version 1.0 [[11](#_ENREF_11)] was used to build the domain architecture of *S. cerevisiae* α-COP (UniProt ID: P53622) (**Figure 1A**). Domain boundaries were obtained by running a conserved domain (CD) similarity search [[12](#_ENREF_12)]. Domain boundary of the RING finger domain was manually delineated.

**References**

1. Altschul SF, Madden TL, Schaffer AA, Zhang J, Zhang Z, Miller W et al. Gapped BLAST and PSI-BLAST: a new generation of protein database search programs. Nucleic Acids Res. 1997;25(17):3389-402.

2. Finn RD, Clements J, Eddy SR. HMMER web server: interactive sequence similarity searching. Nucleic Acids Res. 2011;39(Web Server issue):18.

3. Jaroszewski L, Rychlewski L, Li Z, Li W, Godzik A. FFAS03: a server for profile--profile sequence alignments. Nucleic Acids Res. 2005;33(Web Server issue):W284-8. doi:10.1093/nar/gki418.

4. Soding J, Biegert A, Lupas AN. The HHpred interactive server for protein homology detection and structure prediction. Nucleic Acids Res. 2005;33(Web Server issue):W244-8. doi:10.1093/nar/gki408.

5. Larkin MA, Blackshields G, Brown NP, Chenna R, McGettigan PA, McWilliam H et al. Clustal W and Clustal X version 2.0. Bioinformatics. 2007;23(21):2947-8. doi:10.1093/bioinformatics/btm404.

6. Hall TA, editor. BioEdit: a user-friendly biological sequence alignment editor and analysis program for Windows 95/98/NT. Nucleic acids symposium series; 1999.

7. Li W, Godzik A. Cd-hit: a fast program for clustering and comparing large sets of protein or nucleotide sequences. Bioinformatics. 2006;22(13):1658-9. doi:10.1093/bioinformatics/btl158.

8. Holm L, Sander C. Dali: a network tool for protein structure comparison. Trends in biochemical sciences. 1995;20(11):478-80.

9. Zhang Y, Skolnick J. TM-align: a protein structure alignment algorithm based on the TM-score. Nucleic Acids Res. 2005;33(7):2302-9. doi:10.1093/nar/gki524.

10. Pandit SB, Skolnick J. Fr-TM-align: a new protein structural alignment method based on fragment alignments and the TM-score. BMC bioinformatics. 2008;9:531. doi:10.1186/1471-2105-9-531.

11. Ren J, Wen L, Gao X, Jin C, Xue Y, Yao X. DOG 1.0: illustrator of protein domain structures. Cell research. 2009;19(2):271-3. doi:10.1038/cr.2009.6.

12. Marchler-Bauer A, Lu S, Anderson JB, Chitsaz F, Derbyshire MK, DeWeese-Scott C et al. CDD: a Conserved Domain Database for the functional annotation of proteins. Nucleic Acids Res. 2011;39(Database issue):D225-9. doi:10.1093/nar/gkq1189.
