# Supplementary material for: A novel RING finger in the C-terminal domain of the coatomer protein α-COP
Source: Biol Direct. 2015 Dec 14;10:70. doi: 10.1186/s13062-015-0099-9 (PMC4678705; doi:10.1186/s13062-015-0099-9)
Supplement: Additional file 2: — Supplementary results. Additional supportive evidence in favor of the results presented (DOCX 31 kb) [file 13062_2015_99_MOESM2_ESM.docx]

**A novel RING finger in the C-terminal domain of the coatomer protein α-COP**

Gurmeet Kaur, Srikrishna Subramanian

**Supplementary results**

**Conservation of zinc-chelating residues in the β-sheet domain of α-COP across eukaryotic lineages**

Sequence similarity searches reveal the presence of the α-COP RING finger in a majority of eukaryotic lineages, including fungi, viridiplantae, metazoa, aplicoplexa, choanoflagellida, florideophyceae, bacillariophyta, phaeophyceae, cryptophyta, isochrysidales, saprolegniales, peronosporales, kinetoplastida, trichomonadida, eustigmatophyceae, longamoebia, heterolobosea, dictyostellida, oligohymenophorea, spirotrichea, blastocystis, foraminifera, perkinsida, pelagophyceae, albuginales and fonticula.

The first metal-binding residue of the treble clef is absolutely conserved in fungi, but there are significant variations at the position of the other metal-chelating residues of the RING finger. Fungal species from the genus *Trichophyton* (gi|[607894577](http://www.ncbi.nlm.nih.gov/protein/607894577?report=genbank)), [*Colletotrichum*](http://www.ncbi.nlm.nih.gov/Taxonomy/Browser/wwwtax.cgi?mode=Info&id=5465) (gi|477532069), *Candida* (gi|50289957) preserve all metal-chelating residues of the treble clef motif. Analysis of the sequence from viridiplantae reveals conservation of the metal-chelating residues of the second zinc-binding site in most organisms, except in species from the genus [*Chlorella*](http://www.ncbi.nlm.nih.gov/Taxonomy/Browser/wwwtax.cgi?mode=Info&id=554065) (gi|552845443), *Volvox* (gi|302852603), [*Coccomyxa*](http://www.ncbi.nlm.nih.gov/Taxonomy/Browser/wwwtax.cgi?mode=Info&id=248742) (gi|545374984) and [*Chlamydomonas*](http://www.ncbi.nlm.nih.gov/Taxonomy/Browser/wwwtax.cgi?mode=Info&id=3055) (gi|[159471083](http://www.ncbi.nlm.nih.gov/protein/159471083?report=genbank)). Sequences of the α-COP RING finger from metazoan species, similar to fungi, exhibit variability in the aminoacids constituting both the metal-binding sites. Most organisms belonging to the metazoan lineage have lost their metal-chelating residues at the treble clef metal-binding site and only some, for example, from arthropoda (gi|478259297, 195135403, 340730206), cnidaria (gi|449673896, 156380509), nematoda (gi|402586577, 339254572, 393905469), porifera (gi|340378513), preserve metal-chelating residues at the second metal-binding site of the RING finger. None of the sequences retrieved for chordate species possess all metal-chelating residues at any of the two zinc-binding sites, and thus, would likely not bind metal ions. The sequences belonging to the apicomplexa lineage, from organisms such as *Plasmodium* (gi|574964031) and *Toxoplasma* (gi|237834773) have four absolutely conserved cysteines corresponding to the second metal-binding site and a conserved cysteine corresponding to the first metal-binding residue of the treble clef, whereas other aminoacids variably substitute other three metal-binding residues of the treble clef motif.

**Zinc finger domains in cellular transport pathway proteins**

The ARF-GTPase-activating proteins (ARF-GAPs) that regulate the hydrolysis of the GTP bound to ARF during the Golgi-ER vesicular transport [[1](#_ENREF_1), [2](#_ENREF_2)], have a mononuclear treble clef that is related to the RING finger family [[3](#_ENREF_3), [4](#_ENREF_4)]. The Sec23 and Sec24 proteins of the inner layer of the COPII coat complex possess zinc ribbon domains that have been suggested to play a role in membrane binding and interaction with other subunits of the vesicle cage, respectively [[5](#_ENREF_5)]. Vacuolar protein-sorting 36 (Vps36) of the endosomal sorting complexes required for transport-II (ESCRT-II) also contains a zinc ribbon that mediates interaction with Vps28 of the ESCRT-I complex [[6](#_ENREF_6)]. The binuclear FYVE zinc-binding domain of Vps27, which is a part of the ESCRT-0 machinery helps in tethering to the lipid bilayer by binding to phospholipids [[7](#_ENREF_7)].

**References**

1. Goldberg J. Structural and functional analysis of the ARF1-ARFGAP complex reveals a role for coatomer in GTP hydrolysis. Cell. 1999;96(6):893-902.

2. Nie Z, Randazzo PA. Arf GAPs and membrane traffic. Journal of cell science. 2006;119(Pt 7):1203-11. doi:10.1242/jcs.02924.

3. Grishin NV. Treble clef finger--a functionally diverse zinc-binding structural motif. Nucleic Acids Res. 2001;29(8):1703-14.

4. Krishna SS, Majumdar I, Grishin NV. Structural classification of zinc fingers: survey and summary. Nucleic Acids Res. 2003;31(2):532-50.

5. Zanetti G, Prinz S, Daum S, Meister A, Schekman R, Bacia K et al. The structure of the COPII transport-vesicle coat assembled on membranes. eLife. 2013;2:e00951. doi:10.7554/eLife.00951.

6. Gill DJ, Teo H, Sun J, Perisic O, Veprintsev DB, Emr SD et al. Structural insight into the ESCRT-I/-II link and its role in MVB trafficking. The EMBO journal. 2007;26(2):600-12. doi:10.1038/sj.emboj.7601501.

7. Stahelin RV, Long F, Diraviyam K, Bruzik KS, Murray D, Cho W. Phosphatidylinositol 3-phosphate induces the membrane penetration of the FYVE domains of Vps27p and Hrs. The Journal of biological chemistry. 2002;277(29):26379-88. doi:10.1074/jbc.M201106200.
